# Population genomics: diversity and virulence in the *Neisseria*

**DOI:** 10.1016/j.mib.2008.09.002

**Published:** 2008-10

**Authors:** Martin CJ Maiden

**Affiliations:** Department of Zoology, South Parks Road, Oxford OX1 2PS, United Kingdom

## Abstract

Advances in high-throughput nucleotide sequencing and bioinformatics make the study of genomes at the population level feasible. Preliminary population genomic studies have explored the relationships among three closely related bacteria, *Neisseria meningitidis*, *Neisseria gonorrhoeae* and *Neisseria lactamica*, which exhibit very different phenotypes with respect to human colonisation. The data obtained have been especially valuable in the establishing of the role of horizontal genetic exchange in bacterial speciation and shaping population structure. In the meningococcus, they have been used to define invasive genetic types, search for virulence factors and potential vaccine components and investigate the effects of vaccines on population structure. These are generic approaches and their application to the *Neisseria* provides a foretaste for their application to the wider bacterial world.

## Introduction: microbiology in the post-genome era

The complete genome sequences of hundreds bacterial isolates have been determined, including representatives of the major bacterial pathogens of humans. While this plethora of information has been highly instructive, our capacity to generate data has far exceeded our capacity to interpret it. As this trend continues with next-generation sequence determination technologies, new analytical approaches are needed to exploit these data [1^•^]. A potentially fruitful approach is population genomics, which interprets comparisons of genome or genome derived data in an evolutionary, ecological, and population genetic framework. Much emphasis has been placed on ‘metagenomics’ the ability to study microbial communities without isolating individual genomes, an important consideration when it is possible to isolate fewer than 1% of the bacteria that exist [2].

This article will discuss an alternative and complementary paradigm for population genomics: the study of defined populations of closely related bacteria with distinct phenotypes by comparisons of individual genomes or parts of them. This requires the isolation of a representative sample of a population, normally by culturing the bacteria. Given the extensive diversity of the prokaryotes, this is a demanding task and few exhaustive or even representative bacterial isolate collections exist. Here the genus *Neisseria* will be used to indicate the potential of the multi-genome comparative approach to population genomics.

## The genus *Neisseria* as a model system

Three closely related members of the genus *Neisseria* are of interest medically, exhibiting diverse phenotypes with respect to their interactions with humans. *Neisseria meningitidis*, the meningococcus, is a major cause of meningitis and septicaemia worldwide while *Neissera gonorrhoeae*, the gonococcus, is responsible for one of the most widespread sexually transmitted diseases and *Neisseria lactamica* is a common harmless commensal of human children. All three bacteria are only found colonising the mucosal surfaces, with the meningococcus and *N. lactamica* found in the nasopharynx and the gonococcus associated with the urogenital tract. This is an advantage for population studies as it constrains population sampling: no environmental or animal reservoir need be considered. The lack of good animal models of human disease or infection by these important pathogens has provided further impetus to the exploitation of genomics to investigate their biology [3].

All three organisms have been studied at the population level and large isolate collections are available. The genomes of at least one example of each of the microbiological species have been determined and, at around 2.2 Mbp in size, they are at the lower end of the size range for cultivable bacteria [4–7,8^••^]. Despite being closely related these bacteria are highly diverse. For example, at the time of writing (July 2008) the PubMLST database for *Neisseria*, which catalogues genetically distinct members of the three species as sequence types (STs) based on ‘conserved’ housekeeping genes, listed a total 6814 unique STs (!). This genomic diversity, while posing challenges, can be harnessed to provide power for comparative studies.

## Methods for comparing genomes

Population genomic studies require structured sampling at both the population and the genome level. Obtaining an adequate sample of a bacterial population relies on an understanding of the ecology of the organism and should be further defined by the questions to be asked of the sample collected. For the gonococcus, for example, a collection of disease isolates from a cosmopolitan city can provide a good sample of global genetic diversity. In the case of the meningococcus, however, the collection of isolates solely from cases of invasive disease provides a distorted view of population structure as this organism is an accidental pathogen, with disease not contributing to transmission among human hosts, and distinct genotypes are present in different locales at different times [9^•^]. A more complete picture of this organism comes from the study of isolates obtained from asymptomatic carriage, with the comparison of these isolates with matched isolates from diseased patients instructive in understanding pathogenicity [10].

Comparisons of bacterial genomes can involve the whole genome or representative samples from the genome and may be undertaken with or without reference to complete annotated genome sequences. Direct comparison of genomic DNA from two or more isolates can be performed in various ways, for example representational difference analysis [11,12], and these comparisons have the potential to identify sets of genes that are preset in one isolate but absent in another. Alternatively, sequence variation at a subset of loci can be compared in multiple isolates, as for example in multi-locus sequence typing (MLST) [13]. The latter approach is scalable with existing technology and has been performed on thousands of bacteria in individual studies [14,15^•^] but, of course, only analyses a fraction of the genome.

Gene content and gene diversity can also be established by the comparisons of complete annotated genome sequences, an approach that will become increasingly feasible with newer very high-throughput sequencing technologies. This represents the most exhaustive method but is limited to those genomes that have been completely assembled [4–7]. Partial or not fully assembled genome sequences can also be included in such analyses, which are relatively labour intensive [8^••^]. However, in addition to collecting the data, which shall undoubtedly become much cheaper and faster, analysing complete genomes from tens, hundreds or thousands of isolates is likely to remain a challenge for the foreseeable future.

The construction of microarrays based on annotated sequences enables the presence and absence of genes in many different isolates to be established relatively quickly and cheaply [16–18,19^•^,20,21]. This approach is limited by the sequences used to generate the microarray and does not easily identify sequence variants, being most successful at identifying the presence or absence of genes [22]. For the *Neisseria*, it seems that the ‘pan genome’ is extensive and the construction of an exhaustive ‘pan *Neisseria*’ microarray is probably not achievable [8^••^]. In conclusion, no ideal means of comparing genomes exists at the current time —different approaches are required to address different questions.

## Genetic structure within and among *Neisseria* species

Studies of the *Neisseria* played an important role in the development of bacterial species concepts, being instrumental in establishing the role that recombination plays in shaping bacterial populations. The *Neisseria* are naturally competent for transformation and interspecies and intraspecies recombination has been widely reported at numerous of loci [23]. However, this must be constrained in some way if the convergence of the distinct species, or despeciation [24^•^], is to be avoided. To this end the fate of hybrid genotypes is important. It has been shown, for example, that hybrid penicillin binding protein genes have spread in meningococcal populations, presumably a consequence of positive selection resulting from their higher fitness in the face of the selection pressures imposed by the widespread use of penicillin [25]. By contrast, *N. lactamica* transferrin binding proteins, when acquired by the meningococcus may give a short term within-host advantage but appear to render the hybrids less fit for transmission among hosts, and therefore do not persist [26]. Studies of gene flow among population samples of the three *Neisseria* species show them to be highly differentiated, suggesting that either interspecies recombination is rare or that the hybrids formed are rapidly purged from the population [27]. Whether the limitations to gene flow necessary for the maintenance of these bacterial species [28^•^] are due to selection against hybrids or to biochemical or ecological barriers [29] to gene exchange remains to be determined.

Studies of other pathogens, especially the enteric bacteria, have identified great genome fluidity in terms of gene content [30]; however, this does not appear to be the case within or among the three *Neisseria* species, with the majority of genes shared among the three species [19^•^,31]. Genomic studies have by and large failed to identify major differences that have not been identified previously by more conventional molecular microbiology [17]. Microarray genome hybridisation identified only 20 potential ‘core genes’, mostly encoding hypothetical proteins, that were present in a panel of 48 *N. meningitidis* isolates but absent in *N. lactamica* and *N. gonorrhoeae* in [18]. The most common identifiable differences among *Neisseria* isolates are, intriguingly, restriction modification systems. This may indicate a role for restriction modification systems in limiting gene flow.

An interesting light on the role of recombination in the *Neisseria* has been shed by studies of the distribution of DNA uptake sequences (DUS) in their genomes. These repeated sequences DNA are required for transformation and are present throughout the genome. They are concentrated in regions encoding DNA maintenance and other ‘core’ genes [32] with genome comparisons demonstrating a strong link between recombination and DUS. These observations suggest that DUSs may be important in genome stability [33^••^]. While studies of horizontal genetic exchange have concentrated on the role it plays in generating diversity [34], most recombination will be conservative and homogenising. It is therefore plausible that recombination is in fact primarily a mechanism for genome repair that can occasionally result in generation of diversity, which, even more occasionally, is adaptive. It may be that this repair function is especially important in the *Neisseria*, which lack several DNA repair genes [35]. This would be consistent with restriction modification systems promoting recombination with DNA from very close relatives [36].

## Genetic structure of meningococcal populations

Meningococcal populations, especially those recovered from asymptomatic carriage, are highly diverse with extensive genetic exchange generating novel combinations of existing genes [37–39]. Nevertheless, the population is highly structured into clonal complexes, groups of related meningococci defined in MLST studies by conserved combinations of housekeeping genes. Only a subset of these, the so-called hyperinvasive lineages, are responsible for most diseases [9^•^]. These were first described among isolates from invasive disease [40] and are remarkably stable over time and during geographic spread [9^•^], suggesting that they are resistant to diversification by the high-observed rates of recombination. Short term domination of a recombining population by certain clones can be accommodated, in given locale, by a neutral model with ‘microepidemic’ structure [41], but global distribution over many decades cannot, suggesting that these clonal complexes may be the result of stabilising selection for housekeeping gene combinations [42].

The clonal complexes exhibit medically relevant phenotypes, the most important being differences in their propensity to cause disease, which can be as great as two orders of magnitude [10]. Clonal complexes are also associated with particular repertoires of antigenic variants [43–45], perhaps as a consequence of immune selection acting during carriage [46]—this has obvious implications for the design of vaccines against this highly variable bacterium. The capsule region, encoding the ability to synthesise a polysaccharide capsule, remains the principal difference between carriage and disease-associated meningococci, and indeed between meningococci and gonococci and *N. lactamica*. Capsule type (known as ‘Group’ in meningococci) is also correlated with clonal complex, although this association can change with time [9^•^]. As a virulence determinant even the role of capsule is ambiguous: only five or six of the 12 recognised capsule variants are ever associated with disease and while capsule expression is necessary it is not sufficient for a meningococcus to cause disease.

In principle, the existence of defined genetic types with different phenotypes provides the prospect of identifying genetic traits responsible for those phenotypes by genome-wide association studies [47]. These rely on well-defined and usually quite large isolate collections. A number of such studies have been undertaken but as yet the only new element to be associated with meningococcal disease is a putative phage, identified by whole genome comparisons of disease and carriage isolates [48]. A potential problem with this approach is that the propensity to cause disease is likely to be polygenic and dependent on combinations of genes or even allelic variants of genes also present in less invasive meningococci.

## Some practical applications of population genomics

The development of a comprehensive meningococcal vaccine remains a major research goal. Knowledge of the population structuring of meningococcal protein antigen repertoires has obvious implications in the design of vaccine recipes, potentially limiting the number of variants that need to be included to a manageable number [49]. Meningococcal genome data have also been mined to identify vaccine candidates with ‘reverse vaccinology’ [50] that has yielded novel vaccine candidates currently being exploited in vaccine development programmes. Interestingly, although whole genome mutational approaches have been applied to the meningococcus they too have failed to identify major novel virulence components [51,52], perhaps indicating the weakness of animal models in this area and the polygenic nature of meningococcal virulence. Finally, the effect of the serogroup C conjugate polysaccharide vaccine on the meningococcal population was established by means of a survey of the genotypes carried by teenagers before and after vaccine introduction in 1999, showing that the vaccine was, in large degree, highly effective as a result of very high protection against carriage of one particular genotype (ST-11) associated with both disease and the serogroup C capsule. This study required the characterisation of over 8000 isolates by MLST [15^•^].

## Conclusions

While illustrating the power of population genomics in understanding bacteria by cataloguing their diversity with reference to particular phenotypes, the example of the *Neisseria* also gives glimpse of the scale of the task ahead. This group of closely related bacteria, which are only found in a highly specialised niche, the mucosal surfaces of humans, nevertheless exhibit bewildering level of diversity at the genome level—even in relatively conserved genes. Literally thousands of bacterial genomes need to be collected by carefully structured sampling and analysed by genome-wide approaches to map the diversity of this very limited corner of microbial life and to investigate the complex dynamics within it. Computationally intensive analyses will be required to interpret this diversity and understand the forces that shape it. When these considerations are translated to the much greater diversity present in other, more adaptable, pathogens and then on to free-living bacteria, most of which we have only a rudimentary knowledge, bacterial diversity is both awe-inspiring and humbling. We have only just begun to appreciate the extent of our ignorance of the prokaryotic world of which we form such a small part.

## References and recommended reading

Papers of particular interest, published within the period of review, have been highlighted as:

• of special interest
•• of outstanding interest

## References

[1•] Medini D., Serruto D., Parkhill J., Relman D.A., Donati C., Moxon R., Falkow S., Rappuoli R. (2008). Microbiology in the post-genomic era. Nat Rev Microbiol.

[2] DeLong E.F. (2004). Microbial population genomics and ecology: the road ahead. Environ Microbiol.

[3] Schoen C., Joseph B., Claus H., Vogel U., Frosch M. (2007). Living in a changing environment: Insights into host adaptation in *Neisseria meningitidis* from comparative genomics. Int J Med Microbiol.

[4] Tettelin H., Saunders N.J., Heidelberg J., Jeffries A.C., Nelson K.E., Eisen J.A., Ketchum K.A., Hood D.W., Peden J.F., Dodson R.J. (2000). Complete genome sequence of *Neisseria meningitidis* serogroup B strain MC58. Science.

[5] Parkhill J., Achtman M., James K.D., Bentley S.D., Churcher C., Klee S.R., Morelli G., Basham D., Brown D., Chillingworth T. (2000). Complete DNA sequence of a serogroup A strain of *Neisseria meningitidis* Z2491. Nature.

[6] Bentley S.D., Vernikos G.S., Snyder L.A., Churcher C., Arrowsmith C., Chillingworth T., Cronin A., Davis P.H., Holroyd N.E., Jagels K. (2007). Meningococcal genetic variation mechanisms viewed through comparative analysis of serogroup C Strain FAM18. PLoS Genet.

[7] Peng J., Yang L., Yang F., Yang J., Yan Y., Nie H., Zhang X., Xiong Z., Jiang Y., Cheng F. (2008). Characterization of ST-4821 complex, a unique *Neisseria meningitidis* clone. Genomics.

[8••] Schoen C., Blom J., Claus H., Schramm-Gluck A., Brandt P., Muller T., Goesmann A., Joseph B., Konietzny S., Kurzai O. (2008). Whole-genome comparison of disease and carriage strains provides insights into virulence evolution in *Neisseria meningitides*. Proc Natl Acad Sci U S A.

[9•] Caugant D.A. (2008). Genetics and evolution of *Neisseria meningitidis*: importance for the epidemiology of meningococcal disease. Infect Genet Evol.

[10] Yazdankhah S.P., Kriz P., Tzanakaki G., Kremastinou J., Kalmusova J., Musilek M., Alvestad T., Jolley K.A., Wilson D.J., McCarthy N.D. (2004). Distribution of serogroups and genotypes among disease-associated and carried isolates of *Neisseria meningitidis* from the Czech Republic, Greece, and Norway. J Clin Microbiol.

[11] Tinsley C.R., Nassif X. (1996). Analysis of the genetic differences between *Neisseria meningitidis* and *Neisseria gonorrhoeae*: two closely related bacteria expressing two different pathogenicities. Proc Natl Acad Sci U S A.

[12] Bart A., Dankert J., van der Ende A. (2000). Representational difference analysis of *Neisseria meningitidis* identifies sequences that are specific for the hyper-virulent lineage III clone. FEMS Microbiol Lett.

[13] Maiden M.C. (2006). Multilocus sequence typing of bacteria. Annu Rev Microbiol.

[14] Brehony C., Jolley K.A., Maiden M.C. (2007). Multilocus sequence typing for global surveillance of meningococcal disease. FEMS Microbiol Rev.

[15•] Maiden M.C., Ibarz-Pavon A.B., Urwin R., Gray S.J., Andrews N.J., Clarke S.C., Walker A.M., Evans M.R., Kroll J.S., Neal K.R. (2008). Impact of meningococcal serogroup C conjugate vaccines on carriage and herd immunity. J Infect Dis.

[16] Grifantini R., Bartolini E., Muzzi A., Draghi M., Frigimelica E., Berger J., Ratti G., Petracca R., Galli G., Agnusdei M. (2002). Previously unrecognized vaccine candidates against group B meningococcus identified by DNA microarrays. Nat Biotechnol.

[17] Stabler R.A., Marsden G.L., Witney A.A., Li Y., Bentley S.D., Tang C.M., Hinds J. (2005). Identification of pathogen-specific genes through microarray analysis of pathogenic and commensal *Neisseria* species. Microbiology.

[18] Hotopp J.C., Grifantini R., Kumar N., Tzeng Y.L., Fouts D., Frigimelica E., Draghi M., Giuliani M.M., Rappuoli R., Stephens D.S. (2006). Comparative genomics of *Neisseria meningitidis*: core genome, islands of horizontal transfer and pathogen-specific genes. Microbiology.

[19•] Snyder L.A., Saunders N.J. (2006). The majority of genes in the pathogenic *Neisseria* species are present in non-pathogenic *Neisseria lactamica*, including those designated as ‘virulence genes’. BMC Genomics.

[20] Claus H., Vogel U., Swiderek H., Frosch M., Schoen C. (2007). Microarray analyses of meningococcal genome composition and gene regulation: a review of the recent literature. FEMS Microbiol Rev.

[21] Peng J., Zhang X., Shao Z., Yang L., Jin Q. (2008). Characterization of a new *Neisseria meningitidis* serogroup C clone from China. Scand J Infect Dis.

[22] Swiderek H., Claus H., Frosch M., Vogel U. (2005). Evaluation of custom-made DNA microarrays for multilocus sequence typing of *Neisseria meningitidis*. Int J Med Microbiol.

[23] Hanage W.P., Fraser C., Spratt B.G. (2005). Fuzzy species among recombinogenic bacteria. BMC Biol.

[24•] Sheppard S.K., McCarthy N.D., Falush D., Maiden M.C. (2008). Convergence of *Campylobacter* species: implications for bacterial evolution. Science.

[25] Taha M.K., Vazquez J.A., Hong E., Bennett D.E., Bertrand S., Bukovski S., Cafferkey M.T., Carion F., Christensen J.J., Diggle M. (2007). Target gene sequencing to characterize the penicillin G susceptibility of *Neisseria meningitidis*. Antimicrob Agents Chemother.

[26] Zhu P., van der Ende A., Falush D., Brieske N., Morelli G., Linz B., Popovic T., Schuurman I.G., Adegbola R.A., Zurth K. (2001). Fit genotypes and escape variants of subgroup III *Neisseria meningitidis* during three pandemics of epidemic meningitis. Proc Natl Acad Sci U S A.

[27] Bennett J.S., Jolley K.A., Sparling P.F., Saunders N.J., Hart C.A., Feavers I.M., Maiden M.C. (2007). Species status of *Neisseria gonorrhoeae*: evolutionary and epidemiological inferences from MLST. BMC Biol.

[28•] Fraser C., Hanage W.P., Spratt B.G. (2007). Recombination and the nature of bacterial speciation. Science.

[29] Koeppel A., Perry E.B., Sikorski J., Krizanc D., Warner A., Ward D.M., Rooney A.P., Brambilla E., Connor N., Ratcliff R.M. (2008). Identifying the fundamental units of bacterial diversity: a paradigm shift to incorporate ecology into bacterial systematics. Proc Nat Acad Sci U S A.

[30] Ahmed N., Dobrindt U., Hacker J., Hasnain S.E. (2008). Genomic fluidity and pathogenic bacteria: applications in diagnostics, epidemiology and intervention. Nat Rev Microbiol.

[31] Snyder L.A., Jarvis S.A., Saunders N.J. (2005). Complete and variant forms of the ‘gonococcal genetic island’ in *Neisseria meningitidis*. Microbiology.

[32] Davidsen T., Rodland E.A., Lagesen K., Seeberg E., Rognes T., Tonjum T. (2004). Biased distribution of DNA uptake sequences towards genome maintenance genes. Nucleic Acids Res.

[33••] Treangen T.J., Ambur O.H., Tonjum T., Rocha E.P. (2008). The impact of the neisserial DNA uptake sequences on genome evolution and stability. Genome Biol.

[34] Hanage W.P., Fraser C., Spratt B.G. (2006). The impact of homologous recombination on the generation of diversity in bacteria. J Theor Biol.

[35] Davidsen T., Tonjum T. (2006). Meningococcal genome dynamics. Nat Rev Microbiol.

[36] Claus H., Friedrich A., Frosch M., Vogel U. (2000). Differential distribution of novel restriction-modification systems in clonal lineages of *Neisseria meningitidis*. J Bacteriol.

[37] Claus H., Maiden M.C., Wilson D.J., McCarthy N.D., Jolley K.A., Urwin R., Hessler F., Frosch M., Vogel U. (2005). Genetic analysis of meningococci carried by children and young adults. J Infect Dis.

[38] Jolley K.A., Kalmusova J., Feil E.J., Gupta S., Musilek M., Kriz P., Maiden M.C. (2002). Carried meningococci in the Czech Republic: a diverse recombining population. J Clin Microbiol.

[39] Jolley K.A., Wilson D.J., Kriz P., McVean G., Maiden M.C. (2005). The influence of mutation, recombination, population history, and selection on patterns of genetic diversity in *Neisseria meningitidis*. Mol Biol Evol.

[40] Caugant D.A., Frøholm L.O., Bovre K., Holten E., Frasch C.E., Mocca L.F., Zollinger W.D., Selander R.K. (1986). Intercontinental spread of a genetically distinctive complex of clones of *Neisseria meningitidis* causing epidemic disease. Proc Natl Acad Sci U S A.

[41] Fraser C., Hanage W.P., Spratt B.G. (2005). Neutral microepidemic evolution of bacterial pathogens. Proc Natl Acad Sci U S A.

[42] Buckee CO, Jolley K, Recker M, Penman B, Kriz P, Gupta S, Maiden MC: **Role****of selection in the emergence of lineages and the evolution of virulence in** ***Neisseria meningitidis***. *Proc Natl Acad Sci U S A*, in press. 10.1073/pnas.0712019105 PMC2553036 18815379

[43] Urwin R., Russell J.E., Thompson E.A., Holmes E.C., Feavers I.M., Maiden M.C. (2004). Distribution of surface protein variants among hyperinvasive meningococci: implications for vaccine design. Infect Immun.

[44] Harrison L.H., Jolley K.A., Shutt K.A., Marsh J.W., O’Leary M., Sanza L.T., Maiden M.C. (2006). Antigenic shift and increased incidence of meningococcal disease. J Infect Dis.

[45] Callaghan M.J., Jolley K.A., Maiden M.C. (2006). Opacity-associated adhesin repertoire in hyperinvasive *Neisseria meningitidis*. Infect Immun.

[46] Callaghan M.J., Buckee C.O., Jolley K.A., Kriz P., Maiden M.C., Gupta S. (2008). The effect of immune selection on the structure of the meningococcal opa protein repertoire. PLoS Pathogens.

[47] Falush D., Bowden R. (2006). Genome-wide association mapping in bacteria?. Trends Microbiol.

[48] Bille E., Zahar J.R., Perrin A., Morelle S., Kriz P., Jolley K.A., Maiden M.C., Dervin C., Nassif X., Tinsley C.R. (2005). A chromosomally integrated bacteriophage in invasive meningococci. J Exp Med.

[49] Russell J.E., Urwin R., Gray S.J., Fox A.J., Feavers I.M., Maiden M.C. (2008). Molecular epidemiology of meningococcal disease in England and Wales 1975–1995, before the introduction of serogroup C conjugate vaccines. Microbiology.

[50] Pizza M., Scarlato V., Masignani V., Giuliani M.M., Arico B., Comanducci M., Jennings G.T., Baldi L., Bartolini E., Capecchi B. (2000). Identification of vaccine candidates against serogroup B meningococcus by whole-genome sequencing. Science.

[51] Geoffroy M.C., Floquet S., Metais A., Nassif X., Pelicic V. (2003). Large-scale analysis of the meningococcus genome by gene disruption: resistance to complement-mediated lysis. Genome Res.

[52] Sun Y.H., Bakshi S., Chalmers R., Tang C. (2000). Functional genomics of *Neisseria meningitidis* pathogenesis. Nat Med.

